# In silico evo-devo: reconstructing stages in the evolution of animal segmentation

**DOI:** 10.1186/s13227-016-0052-8

**Published:** 2016-08-01

**Authors:** Renske M. A. Vroomans, Paulien Hogeweg, Kirsten H. W. J. ten Tusscher

**Affiliations:** Theoretical Biology, Utrecht University, Padualaan 8, 3584 CH Utrecht, The Netherlands

**Keywords:** Segmentation, In silico evolution, Axis extension, Posterior signalling, Determinate growth, Bilaterian evolution

## Abstract

**Background:**

The evolution of animal segmentation is a major research focus within the field of evolutionary–developmental biology. Most studied segmented animals generate their segments in a repetitive, anterior-to-posterior fashion coordinated with the extension of the body axis from a posterior growth zone. In the current study we ask which selection pressures and ordering of evolutionary events may have contributed to the evolution of this specific segmentation mode.

**Results:**

To answer this question we extend a previous in silico simulation model of the evolution of segmentation by allowing the tissue growth pattern to freely evolve. We then determine the likelihood of evolving oscillatory sequential segmentation combined with posterior growth under various conditions, such as the presence or absence of a posterior morphogen gradient or selection for determinate growth. We find that posterior growth with sequential segmentation is the predominant outcome of our simulations only if a posterior morphogen gradient is assumed to have already evolved and selection for determinate growth occurs secondarily. Otherwise, an alternative segmentation mechanism dominates, in which divisions occur in large bursts through the entire tissue and all segments are created simultaneously.

**Conclusions:**

Our study suggests that the ancestry of a posterior signalling centre has played an important role in the evolution of sequential segmentation. In addition, it suggests that determinate growth evolved secondarily, after the evolution of posterior growth. More generally, we demonstrate the potential of evo-devo simulation models that allow us to vary conditions as well as the onset of selection pressures to infer a likely order of evolutionary innovations.

**Electronic supplementary material:**

The online version of this article (doi:10.1186/s13227-016-0052-8) contains supplementary material, which is available to authorized users.

## Background

Segmentation, the division of the animal body plan into multiple, repeating units, has fascinated evolutionary and developmental biologists alike. Only the vertebrates, arthropods and annelids display overt body segmentation, while several other clades show intermediate levels of segmentation in only a subset of tissues or organs, a property called metamerism [1–3]. Repetitive patterning is studied in most detail in overtly segmented animals. In these clades, segments are typically laid down in a regular anterior–posterior sequence, via a process involving posterior growth (also called terminal addition) and periodic, sequential generation of segments [3, 4]. A famous exception is the fruit fly Drosophila in which segments are laid down simultaneously across a preformed body axis.

It is currently unresolved why segmented animals mostly display this superficially similar, sequential mode of segmentation. This issue is partly related to the question of whether segmentation was present in the bilaterian ancestor, either as overt segmentation or as metamerism, or rather that it evolved multiple times in parallel in the different lineages [1, 2, 4–13]. Arguments in favour of a single origin of segmentation include the prevalence of sequential segmentation [3, 4]. Studies using ancestral state reconstruction suggest that this mode of segment addition via posterior outgrowth represents ancestral bilaterian properties [9, 14]. In addition, the three segmented lineages have shared genes involved in segmentation, such as Notch, Engrailed and Wnt [4, 8, 15, 16]. Arguments in favour of parallel evolution of segmentation instead put forward that there are also large differences in the genes responsible for segmentation and that the limited observed overlap in gene usage could be explained by parallel recruitment from the limited developmental genetic toolbox [12]. The precise mechanisms of cell division, axial elongation and sequential segmentation also differ substantially between these lineages, ranging from teloblastic growth and stereotyped cell divisions in annelids and some crustaceans [17, 18], to posterior growth zones in most insects and vertebrates [19, 20] with variable roles of cell division versus cell rearrangement [21, 22]. Furthermore, multiple segmentation processes can take place in different body regions or tissue types even within a single organism, each with their own evolutionary origin [23], which further supports (partial) parallel evolution.

Still, independent of whether sequential segmentation evolved once or multiple times, we can ask whether certain factors or conditions may have contributed to this particular evolutionary outcome. Earlier evo-devo simulation studies have demonstrated that sequential segmentation represents a robust evolutionary outcome with high future evolutionary potential [24–26], suggesting evolutionary advantages of this particular segmentation mode. In addition, prior evolutionary events may have generated biases or constraints that influenced the likelihood of the evolution of sequential segmentation. For example, evolutionary comparisons show that a posterior signalling region characterised by caudal, Wnt and FGF signalling predates the origin of the bilaterians [27]. Furthermore, it was recently suggested that posterior growth through terminal addition was already present in the bilaterian ancestor [14]. Thus, we may ask whether these properties have played a decisive role in sending evolution down the path of evolving sequential segmentation.

In the current study we aim to answer these questions. For this we substantially extended a previously used in silico model [26]. Rather than superimposing a particular growth pattern, we incorporate a gene controlling cell division and let evolution determine the type of tissue growth dynamics that arises. Then, by varying whether or not a stable posterior signalling centre is present in simulations, we can investigate the role of such a signalling centre on the type of growth and segmentation that evolves. We thus substantially expanded the degrees of freedom available to the evolutionary process, allowing us to investigate under which conditions sequential segmentation is the most likely evolutionary outcome.

We observe two predominant evolutionary outcomes: sequential segmentation with posterior growth and simultaneous segmentation involving tissue-wide bursts of divisions. We find that the likelihood with which the strategies evolve depends on the type of imposed morphogen dynamics and the strength and timing of an evolutionary pressure for determinate growth. We show that a self-organised posterior signal is more difficult to evolve than a developmental strategy which does not rely on such a posterior centre. From this we conclude that the prior evolution of a posterior signalling centre has played a decisive role in determining the evolution of sequential segmentation. Furthermore, we demonstrate that an evolutionary pressure for determinate growth reduces the likelihood of evolving sequential segmentation. When we apply this evolutionary pressure after sequential growth and segmentation have evolved, a mechanism to stop growing can evolve which is coordinated with the pre-existing sequential segmentation. We therefore propose that the order of evolutionary events is key to inferring the likelihood of particular developmental strategies. Reversing the argument, our work strongly suggests that a posterior signalling zone evolved prior to segmentation and that sequential growth and segmentation evolved prior to determinate growth.

## Methods

### The model

#### General set-up

**Fig. 1:**
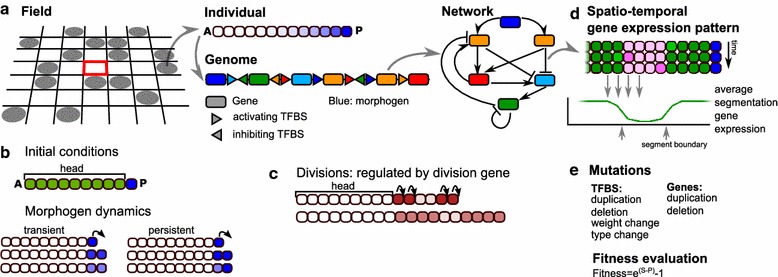
Overview of the model. **a** The developing individuals live on a 2D lattice. Each individual consists of a row of cells. The genome of the individual codes for a network of regulatory interactions, which determines the spatio-temporal dynamics of the proteins within each cell (see **d**). **b** The initial conditions for each new individual at the start of its development. There are a growth zone with high morphogen and a ‘head’ region without morphogen. The morphogen dynamics may vary. Either they are imposed, yielding persistent posterior morphogen (*left*) the morphogen is kept at a high level in the posterior-most cell while decaying in the other cells, or the morphogen can become regulated by the network, so that only the initial conditions are specified (*right*). **c** Divisions are regulated by a division protein; when its level passes a threshold, the cell can divide. Upon division, the level of the division protein in both daughter cells is halved, but not the level of the other proteins. **d** At the end of development, the expression of the segmentation gene is averaged over a number of time steps, and from this the segment boundaries are determined. **e** The mutational operators acting on the genome

We extend an existing individual based model of a population of organisms evolving on a lattice [26] (Fig. 1a). Each organism possesses a so-called pearls-on-a-string genome consisting of genes encoding transcription factors (TFs) and upstream regulatory regions with transcription factor binding sites (TFBS) [28]. At birth, organisms consist of a short one-dimensional row of cells which grows through the course of the individual’s development. An individual’s probability of reproduction (fitness) depends on the number of segments present in its gene expression pattern after a predefined amount of developmental time.

#### Individuals

*Genome, network*

The genome codes for a regulatory network with the genes representing the nodes, and the TFBS as the regulatory links (edges) between nodes (Fig. 1a). These regulatory interactions can be repressive or activating. This network governs gene expression dynamics and hence protein levels. Gene expression dynamics are modelled with ordinary differential equations as shown in Eq. (1):

1 $$\begin{aligned} \hbox {d}G_i/\hbox {d}t & = \frac{\hbox {input}^2}{\hbox {input}^2+1}*E_i-d_i*G_i\nonumber \\ \hbox {input}&= \text {MAX}\left( 0.,\,\sum _{j}{\frac{w_j*G_j^2}{G_j^2+H_j^2}}\,\right) \end{aligned}$$

Transcription of a gene is determined by the summed input of all activating and repressing TFBS regulating this gene, where the influence of each individual TFBS is assumed to depend on TF concentration in a saturating manner. $E_i$ is the maximum expression level of gene *i* and $d_i$ is the degradation rate of the resulting protein; both values can evolve. $w_j$ is the weight determining the strength with which TF *j* influences the expression of gene *i*; this weight is negative (−1) for repressing TF and positive (+1) for activating TF; the sign of these weights is subject to evolution. $H_{j}$ represent the evolvable Hill constants of the TFBS, where the Hill constant corresponds to the level of the TF at which half-maximal activation or repression occurs. The expression of all genes of the same type (see below)  is summed into a single protein level.

*Developmental tool kit and initial conditions* There are 16 types of genes, indicated with a number from 0 to 15.

**Fig. 2:**
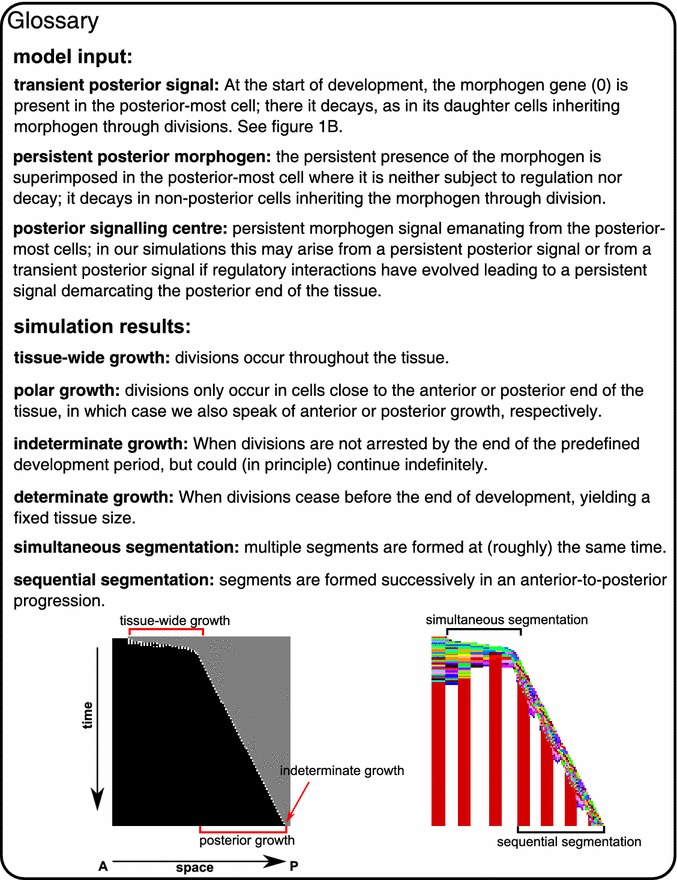
Glossary

**Gene 0 is the morphogen** unless indicated differently, it is not regulated by any of the other genes, thus corresponding to a maternal input. It is kept at a high expression level in the posterior-most cell, while decaying with a predefined rate in the rest of the embryo (Figs. 1b, 2). In a subset of simulations instead, this high posterior expression is only used as an initial condition and is thus not automatically maintained in the posterior-most cell, and the gene may become regulated by other genes.

**Gene 1 and gene 2 are signalling genes**, responsible for direct, membrane bound cell–cell signalling (similar to e.g. Delta–Notch signalling). This direct cell–cell signalling is implemented as follows: if a gene has TFBS of type 1 or 2 in its upstream region, the expression of that gene in a particular cell is regulated by the levels of protein type 1 or 2 in its directly neighbouring cells, while its own intracellular levels of these proteins have no impact on the expression of that gene but only on that of genes in neighbouring cells. If cell–cell signalling is switched off in a simulation, genes 1 and 2 function as normal genes.

**Gene 4 is the division gene** when it is highly expressed (protein level >80. a.u.), the cell may divide with high probability ($p=0.975$). Upon division, the level of only the division gene is halved in the resulting two daughter cells (Fig. 1c).

**Gene 5 is the segmentation gene**, whose final pattern of gene expression along the body axis determines the fitness of the organism.

Individuals start their development with a short row of 10 cells, where the posterior cell forms the primordial “growth zone” in which the morphogen level is high; in the remaining nine cells (the “head” that is assumed to have evolved prior), the morphogen is absent (Fig. 1b). At the start of development genes 6 and 7 are uniformly expressed in the zygote, while other genes have an initial expression level of 0. Throughout development, the protein levels are updated according to the network (Eq. 1).

*Fitness evaluation* At the end of development (after a fixed number of time steps), the number of well-formed segments determines an individual’s fitness. A segment is defined by the average expression pattern of the segmentation gene over the last 20 or 40 developmental steps (Fig. 1d). This averaging helps ensure the evolution of temporally stable segmental patterning, as it will not reward oscillatory segmentation that fails to converge on a constant spatial pattern (as occurred in [25]). Segments should be at least seven cells wide, and boundaries between segments should consist of a clear transition of the expression of the segmentation gene from a high to a low level, or vice versa, within five cells (similar to earlier definitions [24, 26]. The number of too narrow segments is subtracted from the number of well-formed ones, reducing the fitness. To further ensure stability of the final developmental pattern, we apply an additional fitness penalty for the amount of variance of the pattern from the average (pattern instability) within the final 20 developmental steps.

In a subset of simulations, some fitness can also be obtained by reaching a target tissue size. This fitness bonus is independent from the number of segments, enabling sequential as well as simultaneous evolution of tissue size and segmentation. We also apply some penalties unrelated to the segments. First, we require that at least one gene of each type is present in the genome; if this requirement is not met, the individual is not allowed to reproduce. Second, a penalty is applied when the individual grows larger than the target final tissue size. Finally, small fitness penalties are used for gene and TFBS numbers in order to prevent excessive genome growth. The fitness then becomes $e^{F}-1$.

2 $$\begin{aligned} F&=\text {nr good segments}\nonumber \\&\quad - \text {nr narrow segments}\nonumber \\&\quad + \text {proximity to target size}\nonumber \\&\quad - G*\text {gene nr}\nonumber \\&\quad - T*\text {TFBS nr}\nonumber \\&\quad - U*\text {nr unstable cells} \end{aligned}$$

See Table 1 for all parameter values.

**Table 1:** Parameter values

Parameter	Values	Remarks
*General*		
Grid size	30 × 30	
Evolutionary time steps	50,000	
Death rate	0.5	
Initial # agents	50	
*Development*		
Developmental time steps	240	The number of integration steps
Integration step size	1	Fourth-order Runge–Kutta integration
Morphogen decay rate	0.2	Only for persistent posterior morphogen of which 9 form the head
Initial tissue size	10 cells	
*Gene and protein dynamics*		
Gene product decay rate	0.05–0.9	
Hill constant of the TFBS	10–400	
Gene transcription	10–100	
*Mutational dynamics*		
Nr of gene types	16	
Gene duplication	0.006	Note that with the gene, also its TFBS is duplicated
Gene deletion	0.009	
TFBS weight change	0.001	
TFBS type change	0.001	
TFBS duplication	0.0015	
TFBS deletion	0.004	
TFBS innovation	0.001	Spontaneous emergence of new TFBS
*Fitness*		
G: penalty per gene	0.0001	
T: penalty per TFBS	0.00001	
Bonus for final tissue size	0 or 0.1	Per cell added by division
Target size	110 cells	
Penalty for exceeding target size	1	For each cell more than target size
Control period	20 steps	Period over which gene expression stability and sometimes number of late-stage divisions is measured
U: expression variance penalty	0.1	Penalty per cell that has a variance in segmentation gene level >5.0 during the control period

#### Evolution

*Initial conditions, mutations and simulations* The population is initialised with 50 genetically identical individuals. The population resides in a grid of size 30 × 30, imposing a maximum to the population size of 900 individuals. The genome of the initial individuals contains a single copy of each gene, in randomised order and with an average of 2 TFBS of random type upstream. Individuals compete for reproduction into a neighbouring empty spot. Those with a higher fitness have a larger probability of being selected. Specifically, an individual’s chance to reproduce is proportional to its fitness divided by the sum over the fitness of itself and the other individuals neighbouring the empty position. Death occurs with a constant probability. Upon reproduction, the genome is mutated via duplications and deletions of both genes and TFBS (Fig. 1d). TFBS may also mutate their type (which protein binds), weight (activating or repressing) and Hill constant, and new TFBS may appear de novo as an innovation. Genes may mutate their maximum activation level *E* and decay rate *d*. Gene duplication also copies the associated TFBS and results in multiple genes of the same type. The expression of all genes of the same type therefore contributes to the expression level of a single protein. Note that since there are no mutations that change the gene type, gene duplication cannot be followed by subsequent divergence in our model.

## Analysis

For each set of model settings and parameter values, we run 50 simulations. Each simulation yields one particular growth and segmentation strategy with only minor variations within the population. Therefore, we only assess one fit individual per simulation. We consider a simulation successful when the fittest individuals at the end of the simulation can generate more than three segments.

**Fig. 3:**
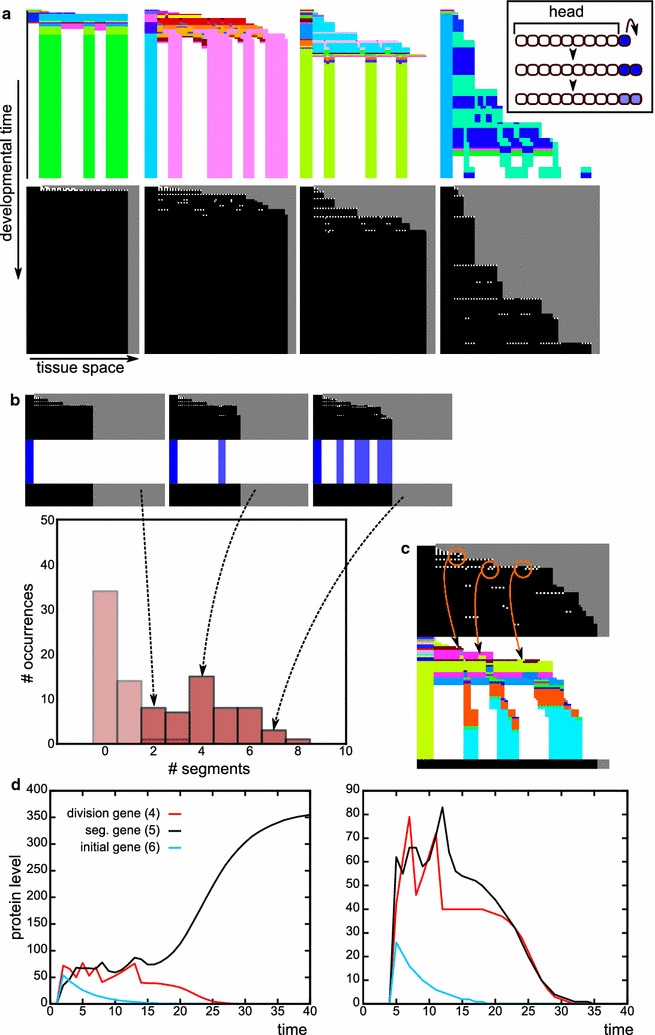
Transient posterior signal without CCS yields only simultaneous segmentation. **a** Space–time plots of successfully evolved individuals, who mainly differ in the timing and number of tissue-wide division bursts. The right-most case only occurred once. The *colour coding in the top row* indicates cell type (based on the levels of all proteins); the *white dots in the bottom row* indicate new (just-divided) cells (see also “Methods”). *Inset* The initial conditions of the morphogen dynamics used in these simulations. The head cells do not divide. The posterior-most cell has high morphogen concentration, which is inherited by its daughters. The morphogen gene can be regulated by the evolving network, just like any other gene, but is not regulated initially. **b** The development of evolved individuals is not robust. The *histogram* depicts the number of bands generated when the development of a single evolved individual is repeated 50 times (see “Methods”). *Lighter bars* indicate the number of too short segments. Examples of the resulting development shown above with space–time plots of the divisions, with as *inset* the expression pattern of the segmentation gene. **c** Division timing plays a role in determining segment position. Cells which by chance happened to divide a bit later (*circled in orange*) form a lineage with high expression of the segmentation gene. **d** Time plots that show the dynamics in a high-segmentation gene stripe (*left*) and a low stripe (*right*). Note the changes in concentration of the division gene due to divisions

*Space–time plots* We use space–time plots as a first impression of the developmental mechanisms that evolved. Because we simulate 1D tissues, we can simply place snapshots of the tissue at many consecutive time points below each other, while keeping the position of the head fixed at the anterior. We display two types of space–time plots; in one, we denote the cell type of each cell with a colour, which represents a unique combination of gene expression values; in the other, cell divisions are depicted by making the newest cells white (in a division, we always consider the posterior daughter cell as the newest, so the anterior daughter stays black), see for example Fig. 3.

*Pruning* Because an evolved genome consists partly of redundant interactions, we prune the genomes via a repeated process of removing genes and binding sites in the genome, while keeping the tissue size and final spatial expression pattern of the segmentation gene the same [26]. This makes it easier to analyse the network by eye and identify the role of the special genes such as the division gene and the segmentation gene. All networks depicted are pruned.

*Assessment of phenotypic variability* We have only one source of noise in our simulations, namely the small probability that a cell with high division gene expression does not immediately divide. Still, this noise may cause some variability in the number of segments that are formed, even if the genome remains exactly the same. We call this phenotypic variability and assess this by repeating the development of an evolved individual 50 times and counting the number of good and malformed segments formed each time. We display the results of this repeated development in a histogram, where we indicate the number of malformed segments with lighter bars. When an individual evolved a mechanism that makes the same number of good segments in 40 or more times out of 50 repeated developments, we call it “developmentally robust”. Note that this differs from the probability of breeding true (mutational robustness): we do not assess how mutations influence the likelihood of producing the same number of segments, as done in [29].

In Table 2, we display the number of times certain segmentation strategies evolve, and the average number of segments made per strategy. We take the phenotypic variability of an individual into account by averaging number of segments it makes during the repeated development mentioned above. These average numbers for single simulations are then averaged over all simulations in which the same developmental strategy evolved.

**Table 2:** Evolved developmental strategies

Simulation set	Simultaneous		Sequential		Other		Failed
*Transient posterior morphogen*							
No CCS	39 (Fig. 3; Additional file 3: Figure S3)	*3.2*	0		0		11
No CCS, no noise	0		0		0		50
With CCS	34 (Fig. 4; Additional file 1: Figure S1)	*6.8*	2 (Fig. 4)	*10.0*	3	*9.1*	11
With CCS, no noise	32	*8.4*	1 (Additional file 4: Figure S4)	*13*	4	*9.3*	13
*Persistent posterior morphogen*							
No CCS	15 (Fig. 7; Additional file 3: Figure S3)	*6.4*	31 (Figs. 6, 7; Additional file 4: Figure S4)	*12.1*	0		4
With CCS	15	*6.9*	22	*12.3*	7	*9.1*	6
Tissue size selection	6 (Fig. 7)	*6.4*	31 (Fig. 7)	*12.7*	8	*10.9*	5

Left number: number of simulations yielding this mechanism (figure number). Right number (italic): average number of segments generated with this mechanism

*Division gene dynamics* In some figures, we display the dynamics of the division gene in the posterior-most cell (containing a high morphogen level). These dynamics result from regulation of the division gene by other genes and from halving of the division gene upon division. In order to see the effect of regulation more clearly, we also show the network dynamics when the halving of the division gene is left out.

## Modelling choices

Body axis segmentation is, like most developmental patterning, a complex phenomenon involving processes ranging from the subcellular to the organism level scale. In this study we simulate the evolution of body axis segmentation and therefore need to simulate development in a population of individuals across many generations. To keep our model tractable both in terms of simulation time and for analysing the evolutionary trajectories of the developmental processes, we substantially simplify the developmental processes in our model relative to real-world developmental processes, but incorporate those properties we deem necessary for studying the evolution of segmentation. Below we detail the three major simplifications, their potential consequences, and why we think these simplifications are justified.

The most obvious simplification is the 1D nature of the model, so that cell divisions automatically lead to an elongated body axis regardless of where they occur in the tissue basically assuming that the axiality is already defined. In reality, developmental patterning occurs in a 2D or 3D tissue, where complex symmetry-breaking events during early development are essential for setting up the anterior–posterior and dorsal–ventral axis. Furthermore, animal axis elongation often involves cellular motility and adhesion properties that are not included in this model. This limits the self-organisation potential of the developmental processes evolving in our model. However, since symmetry breaking is an ancestral property preceding bilaterian evolution [27], we can safely assume that it already existed before segmentation evolved.

We model a cellularised environment in which morphogen gradients are set up through decay dynamics, and signalling is limited to direct receptor–ligand type cell–cell signalling. Neither the morphogen nor any of the other gene products are subject to diffusion. Diffusion plays an important role in Turing-type patterning [30] and in setting up the morphogen gradients dictating early Drosophila segmentation [31]. Still, in Drosophila genes downstream of the morphogen gradients do not require diffusion, and so far no Turing patterns have been found to underlie animal segmentation. Segmentation usually takes place in a cellularised environment, in which the role of diffusion is necessarily restricted to short distances or combined with other gradient establishing mechanisms such as slow mRNA and protein decay [32]. Thus, we are confident that we do not exclude any major real-world segmentation mechanisms from evolving in our model and that leaving out diffusion is justified.

Finally, our model only incorporates gene expression regulation through combinatorial TF regulation on a single regulatory region, ignoring several other factors that may influence gene expression. In vertebrates for instance, the timing of Hox gene mediated axial patterning, and its coordination with segmentation, involves chromosome looping, epigenetic histone and DNA modifications, and cluster level gene regulation [33]. Still, the goal of our study is to explore the evolution of gene expression dynamics, rather than to mimic how these dynamics are precisely regulated. The transcription factor-based regulatory network has sufficient degrees of freedom to allow the evolution of the diverse gene expression dynamics (such as oscillations) that underlie real-world segmentation processes, while supporting computational efficiency and analytical tractability.

## Results

### Evolutionary strategies with transient posterior signal

To investigate the relevance of the prior existence of a stable posterior signalling centre and the morphogen gradients emanating from it for the evolution of posterior growth and sequential segmentation, we performed simulations that do and simulations that do not superimpose the existence of such a signalling centre. We start with an exploration of evolving segmentation strategies in the absence of a superimposed morphogen gradient (Fig. 3a, inset). Instead, we assume transient expression of gene 0 (the “morphogen”), restricted to the posterior-most cell of the embryo, and subject to decay in all cells. As a consequence, this gene will have the same level in the posterior cell as in all cells that descended from it and information on tissue polarity becomes quickly diluted. Under these conditions, a stable posterior signalling centre would have to evolve from scratch by evolving regulation of this transient signal to generate a stable posterior morphogen gradient (rather than being automatically present [24, 26]). Alternatively, a segmentation mechanism could evolve which does not rely on a persistent posterior signal.

We perform four sets of 50 simulations: with/without cell–cell signalling (CCS) and with/without division noise (first four rows in Table 2). The simulations without CCS and without noise form a negative control group which does not have any symmetry-breaking mechanism: indeed, segmentation never evolves. In the remaining sets with either noise, CCS or both, segmentation does evolve. The vast majority of successful simulations (yielding more than three segments) evolves a segmentation strategy in which the tissue grows via one or more short-lived tissue-wide burst of divisions (Fig. 3a). The segments all appear roughly at the same time after the burst of divisions; we call this simultaneous segmentation.

In the absence of CCS and presence of division noise, this simultaneous segmentation mechanism typically yields high phenotypic variability, often generating few segments and only occasionally producing many segments (e.g. Fig. 3b, see “Methods”). The segments are often irregular in size, with some much wider than others. The segmentation mechanism uses the stochastic delay of division in a few cells early in development, which changes the dynamics in those cells sufficiently to differentiate them from their neighbours (Fig. 3c, d). This mechanism therefore does not resemble Drosophila-type simultaneous segmentation but rather reflects the fact that the evolutionary process is free to evolve any possible growth and patterning modes.

**Fig. 4:**
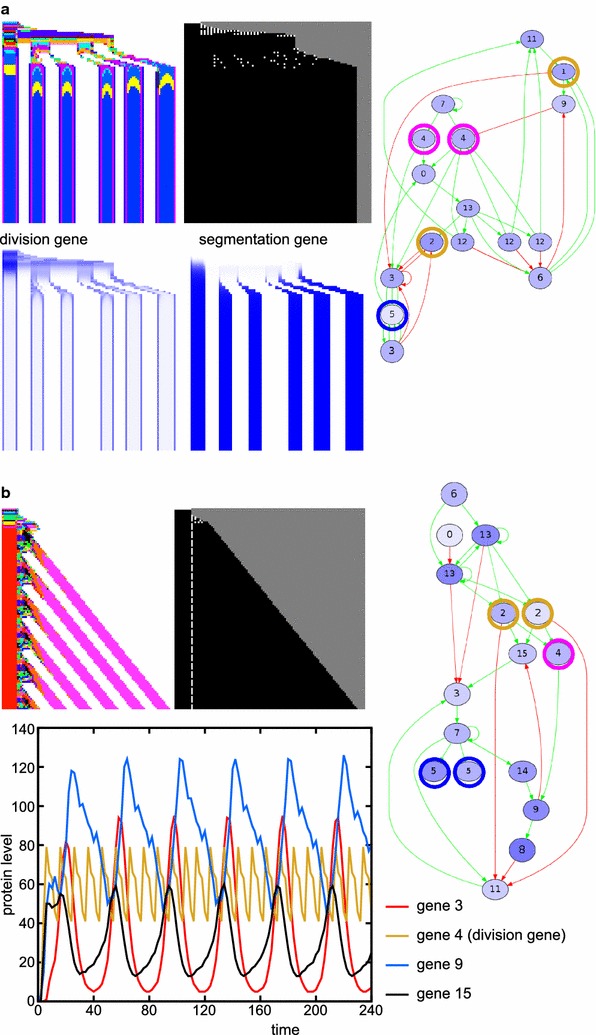
With CCS, different segmentation mechanisms can evolve (transient signal, CCS). **a** This individual uses cell–cell signalling at the boundaries of an emerging segment to split the segments into two. The segmentation gene and the division gene are maintained only in the two boundary cells of this primordial segment, because they receive different inputs from their neighbours. The division gene then generates a new burst of divisions in that cell, expanding the single cell into a new segment. This mechanism piqued our interest because it superficially resembles the splitting of the *odd* stripes in *Tribolium* [42]. It provides an alternative to Turing-like wave splitting in growing media [34], which uses diffusive signalling over longer distances, while this segment-splitting mechanism uses only direct CCS. Another difference is that in the Turing mechanism, the wave splitting results from growth, while here segment-splitting directs divisions. Although cell divisions are thought to play a minor role in the axis extension of *Tribolium*, tissue-wide divisions have been observed that could support the segment-splitting mechanism we find here [22]. In *Tribolium*, however, segment addition happens sequentially, while segment splitting here occurs in simultaneously generated segments. Furthermore, the mechanism in *Tribolium* is distinctly asymmetric: the secondary stripe that splits off is considerably narrower than the primary stripe. It thus remains an open question which mechanism causes segment doubling in *Tribolium*: Turing-like, the mechanism described here, or an as yet unidentified mechanism. **b** This individual uses signalling cues emanating from the static head to stimulate divisions in the cell adjacent to the head. The *graph* depicts the gene expression oscillations that occur in this cell, which subsequently pattern the tissue. In the networks, the division gene is *circled in magenta*, the segmentation gene in *blue* and the signalling genes in *yellow*

In the presence of CCS instead, simultaneous segmentation does not require cell division noise: 37 out of 50 simulations with CCS and without noise evolve segments, while 39 simulations with both CCS and noise yielded segmentation (see Table 2). In simulations with CCS, the average number of segments is doubled compared with the simulations without CCS. Moreover, 13 out of the 39 successful simulations with noise and CCS yield low phenotypic variability, meaning that they are able to make the same number of segments in more than 40 out of 50 repeated developments; we call this developmentally robust (Fig. 4a). Six of the simulations with CCS evolve simultaneous segmentation which uses cell–cell signalling to split developing segments in two (Fig. 4a). This is an alternative to the wave-splitting mechanism observed in Turing pattern systems [34], as the evolved segment-splitting mechanism only relies on signals from direct neighbours rather than feedbacks between diffusive substances. In the presence of CCS, we also find the rare evolution of polarised growth: in two cases the head region is used as a signalling centre for divisions and gene expression oscillations (Fig. 4b). One simulation with CCS and without division noise evolves divisions that are restricted to a broad posterior zone, from which a number of segments appears sequentially (Additional file 2: Figure S2). This mechanism uses signalling from the formed segments to an undifferentiated zone to initiate localised division bursts which then yield new segments.

Altogether, without a superimposed posterior morphogen gradient we obtain a nearly 100 % bias towards simultaneous growth and patterning, with the rare appearance of polarised growth. We therefore next test whether a polarised growth dynamics evolves more frequently if we select for tissue size but not for segmentation, thereby reducing the complexity of the selection target.

**Fig. 5:**
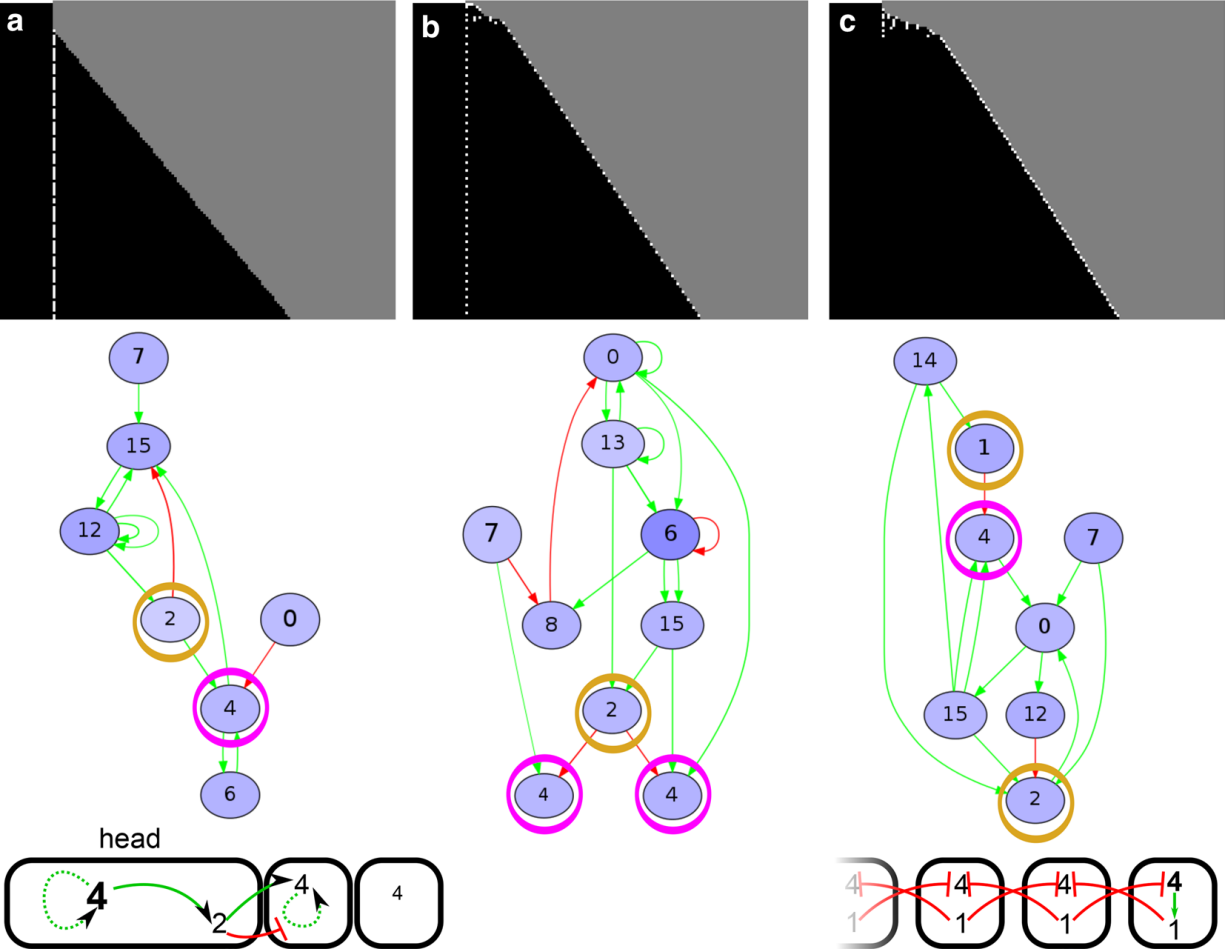
Selection only for tissue size occasionally yields anterior or posterior growth (transient signal, CCS). Examples of individuals which evolved anterior growth (**a**), posterior growth (**c**) or a combination of the two (**b**) in the absence of selection for segments (as described in the main text). **a** Anterior growth exploits the fact that the head does not express gene 0 (morphogen) and does not divide; therefore, it accumulates division protein (4). The head thus functions as a signalling centre. **c** Posterior growth uses the fact that the posterior-most cell has only one neighbouring cell and thus receives less cell–cell signalling. In the networks, the signalling genes 1 and 2 are *circled in yellow* and the division gene in *magenta*

By only selecting for tissue size, the majority of simulations still evolves tissue-wide division bursts as with simultaneous segmentation. We observe anterior growth (with or without initial division burst) in six out of 50 simulations, posterior growth in four cases (in two of which posterior growth is combined with a large initial tissue-wide division burst) and a combination of anterior and posterior growth in two out of 50 simulations (Fig. 5). In these cases, divisions are restricted to the posterior cell because it has only one neighbouring cell and thus receives less inhibitory signal, and a morphogen gradient never evolves. Polarised growth on one end of the tissue thus seems a rare evolutionary outcome, given its low frequency even for a simpler selection target.

Our results show that without a superimposed posterior morphogen gradient, evolution is unlikely to result in posterior growth and sequential segmentation.

### Evolutionary strategies with persistent posterior signal

Next, we performed two sets of simulations with a persistent posterior signal, in the form of a superimposed posterior morphogen gradient: one set with and one without cell–cell signalling, and both with division noise (Table 2). To achieve this, the posterior-most cell now receives a morphogen that is subject to decay in all cells except this posterior-most cell. In these simulations, we find two qualitatively different strategies. The majority of simulations (53 out of 100) evolves a posterior growth zone combined with sequential segmentation, while the tissue-wide burst with simultaneous segmentation observed in the previous section is now less common (30 out of 100). In the simulations with CCS we also find combinations of the simultaneous and sequential mechanism, where the first few segments are generated using a simultaneous mechanism and the remaining segments arise through posterior growth combined with gene expression oscillations (5 simulations).

**Fig. 6:**
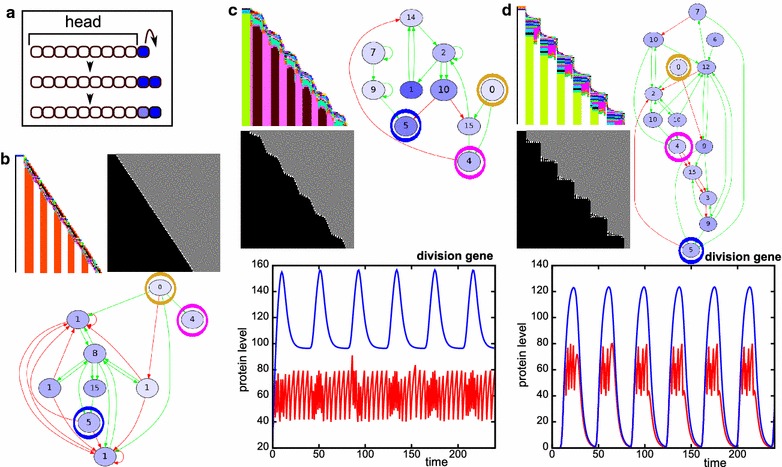
Three different types of sequential segmentation can evolve (persistent signal, no CCS). **a** In these simulations, the morphogen is highly expressed in the posterior-most cell. If that cell divides, morphogen expression is maintained in the posterior daughter, and its level decays in the other cells. **b**–**d** Space–time plots, networks and division gene dynamics of different types of sequential segmentation. In the networks, the morphogen is *circled in yellow*, the division gene in *magenta* and the segmentation gene in *blue*. In the *graphs*, the division gene dynamics are depicted only for the posterior-most cell, with high morphogen level. The *red line* shows the network dynamics if halving of the division protein due to divisions is taken into account. The *blue line* depicts the dynamics if the network is run without halving the division protein once it reaches the division threshold. **b** A smoothly growing individual. Note how the division gene is only regulated by the morphogen. **c** “Wavy” posterior growth. The growth zone keeps dividing, but sometimes its daughters also divide. Note the oscillating expression of the division gene in the posterior-most cell. **d** “Stair-like” posterior growth. The division gene strongly oscillates and is therefore regularly low even in the posterior cell

Often, the evolved sequential segmentation mechanisms are very developmentally robust with only small phenotypic variations, and they yield a large number of segments with a regular pattern (Table 2; Fig. 6). Typically, gene expression oscillations in the growth zone are used to pattern the segments, resembling the mechanism in vertebrates and arthropods. We distinguish three common variations, differing in the distribution of divisions in space and time (Fig. 6b–d). When growth proceeds smoothly, the division gene is only regulated (directly or indirectly) by the morphogen (Fig. 6b). Other variants of posterior growth and sequential segmentation show a wavy or even stair-like growth pattern, reflecting non-continuous, burst-like division dynamics of the posterior growth zone. In these cases the division gene itself oscillates, with low amplitude in the wavy pattern or high amplitude in the stair-like pattern (Fig. 6c, d). These oscillations are caused by regulation of the division gene by other genes in the network that are part of the segmentation oscillator. In Additional file 4: Figure S4 we discuss some non-robust cases of sequential segmentation; there, the division gene is itself a part of the oscillator, making the oscillator sensitive to the stochastic nature of the divisions.

In the absence of CCS, a posterior morphogen gradient improves the developmental robustness of the evolved simultaneous patterning mechanisms (examples in Additional file 3: Figure S3; see Table 2). Cells may use differences in morphogen concentration rather than the differences arising through stochastic cell division for segmental patterning (in fact, now division noise is the source of phenotypic variability, not the patterning mechanism). While adding CCS to simulations with transient posterior morphogen decreased phenotypic variability, in the presence of persistent posterior morphogen no further improvement was observed upon addition of CCS.

So far, we selected for increasing numbers of segments, thus only implicitly selecting for tissue growth. However, it can be hypothesised that body axis elongation—even in the absence of subdivision into segments—already confers an evolutionary advantage. We therefore compare the previously described simulations in which we selected only for segmentation (the set without CCS), to simulations in which we independently select for both axial elongation (to a particular target size) and segmentation. While the number of simulations yielding sequential mechanisms is the same (31), we now find that eight simulations yield the combined simultaneous and sequential strategy, and only six simulations yield fully simultaneous segmentation. Thus, the bias towards sequential segmentation has become somewhat stronger.

**Fig. 7:**
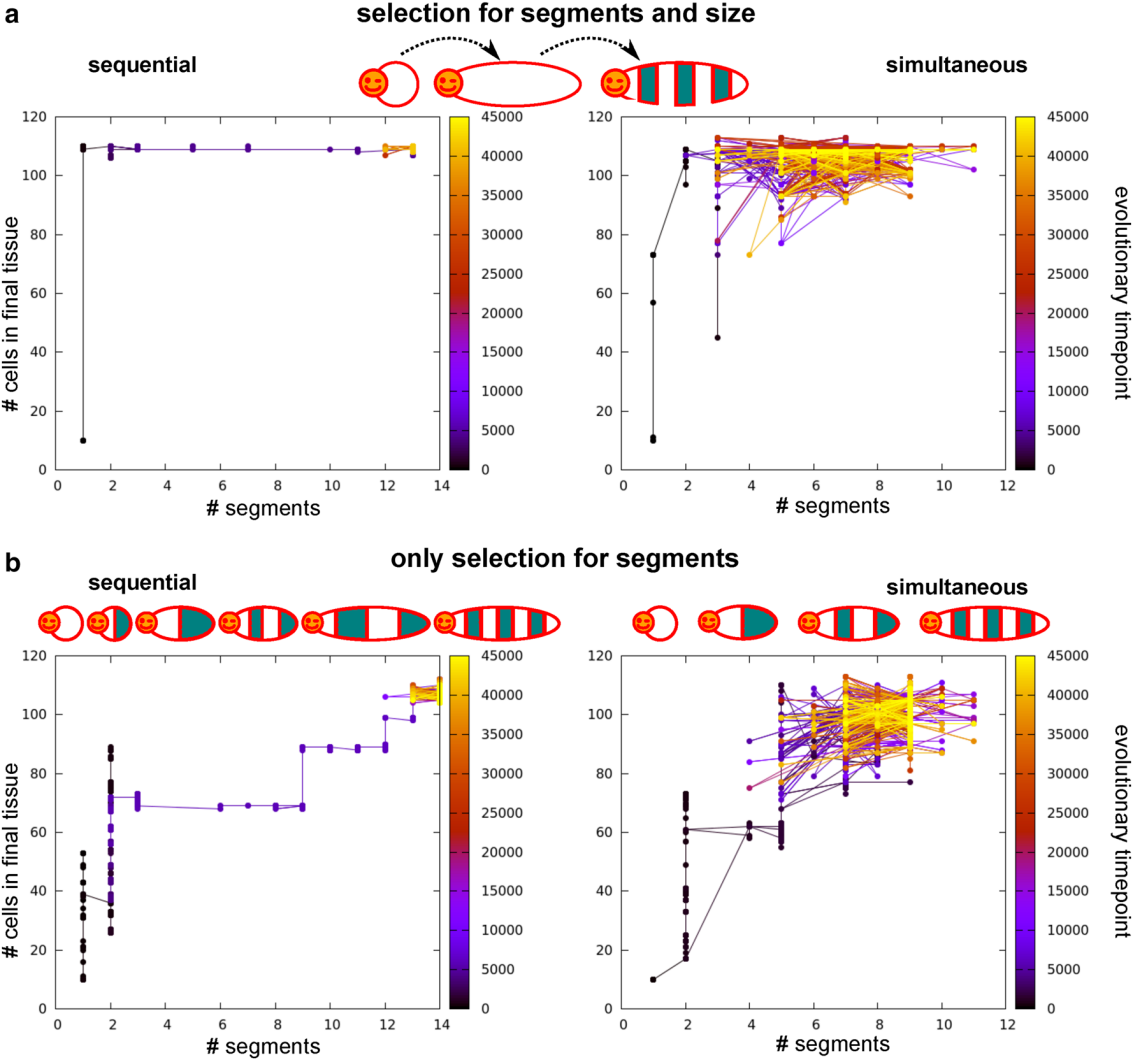
The order of evolutionary events differs between simulations with and without selection for axial extension (persistent signal, no CCS). *Graphs* depict the evolution of final tissue size and segment number for one simulation. *Left column* sequential segmentation, *right* simultaneous segmentation. The *colour of the nodes* indicates the evolutionary time point. **a** Evolutionary trajectories when selecting for axial extension and segmentation: tissue size evolves first to target size before segmentation evolves. **b** Only selection for segments. For sequential segmentation, growth happens in phases. First the available tissue evolves to be filled with segments before tissue size increases further. For simultaneous segmentation, tissue size and number of segments evolve concurrently, but the process is noisy. Note the increased robustness towards the end of the simulation

The evolved developmental mechanisms look similar between the set with and the set without selection for axial elongation, but we find that the evolutionary trajectories that lead to these strategies differ markedly between the sets (Fig. 7). When we select for both axial extension and segmentation, in all simulations we first see the evolution of body axis extension to obtain a tissue close to the target size and subsequently the evolution of a subdivision of the body axis into more and more segments (Fig. 7a). If instead selection is only on segment number, we see differences in the evolution of tissue size between simultaneous and sequential segmenters (Fig. 7b). In the simultaneous case, tissue size increases concurrently with segment number, although the evolutionary sequence is erratic due to the high phenotypic variability of the segmentation mechanism. At later evolutionary time points, we observe a decrease in the variation in tissue size and segment number (Fig. 7b). In the case of sequential segmentation, we instead observe a repeated sequence of first evolving a certain tissue size and subsequently evolving the subdivision of this tissue with an increasing numbers of segments (Fig. 7).

We conclude that, of the possible evolutionary options to segment a tissue, posterior growth coupled to sequential segmentation has a higher potential to be a robust patterning mechanism and is capable of generating more and more regularly shaped segments. In addition, when incorporating an ancestral posterior signalling centre involved in body axis polarity, it is also the most likely evolutionary outcome. This likelihood slightly increases when body axis extension evolves prior to segmentation.

### Evolving determinate growth

From the previous section, it is clear that posterior growth with sequential segmentation is the most successful of the possible developmental strategies: it evolves more often, it is able to form many and regularly shaped segments, and it has the potential to be developmentally robust. So far, we did not take into account that the evolved sequential segmentation mechanisms do not terminate growth at the end of development. Instead they evolve a growth rate that is tuned to allow them to grow to the target size within the constant, superimposed duration of development. If this duration of development were to be extended, larger individuals with a larger number of segments would automatically arise. While there are indeed bilaterian animals (like many annelids) which do continue growing indefinitely [18], most animals stop growing and making segments, for instance vertebrates and insects have a determinate number of segments and roughly determinate growth. We therefore decided to include selection for determinate growth, by applying a fitness penalty for division during the last 20 time steps (no CCS). (Note that the definition applied here for determinate and indeterminate growth is somewhat different from definitions used elsewhere [35]. See also the Fig. 2).

**Fig. 8:**
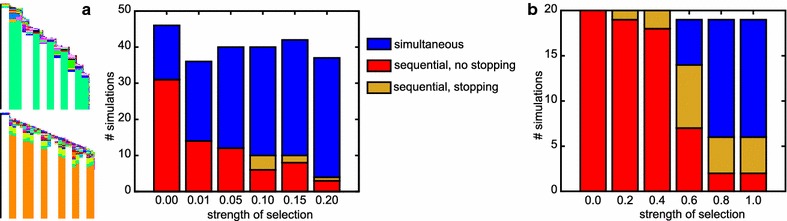
The time of onset of selection for stopping growth influences evolutionary outcome (persistent signal, no CCS). **a** The frequency with which simulations evolve simultaneous segmentation increases with stronger selection pressures to stop growing. Only rarely do individuals evolve with determinate sequential segmentation. **b** When the selection pressure to stop growing is added after segmentation has evolved, sequential segmentation with determinate growth more frequently evolves. The number of simulations which switch to simultaneous growth does still increase with increasing selection pressure. Twenty sequentially growing individuals were allowed to continue evolution with the added pressure. The selection pressure required to effect a change is higher; the period over which divisions are penalised is now also 40 steps instead of 20

With increasing strength of this evolutionary pressure, a larger fraction of simulations yields simultaneous growth and segmentation, until the bias is completely reversed (Fig. 8a). In a subset of simulations stair-like sequential growth evolves, which allows for sequential growth while circumventing the fitness cost of late-stage divisions. Only very rarely (max 4 out of 50) does a simulation yield sequential segmentation with a mechanism that leads to the controlled halting of growth (Fig. 8a, example space–time plots).

We reason that by applying the selection for determinate growth from the start of the simulation, we implicitly select for simultaneous growth, which is indeed determinate. To prevent this bias, sequential segmentation would have to have evolved before the appearance of this selection pressure. It also seems biologically reasonable to assume that determinate growth is a secondary trait: when comparing segmented animals with indeterminate and determinate growth, it seems that at least in arthropods the clades with determinate growth have more evolutionary derived, complex body plans. We speculate that determinate growth becomes more important upon evolution of segment specification, where e.g. locomotive appendages are limited to trunk segments and the abdomen is unsupported. To test the idea of secondary selection for determinate growth, we extract individuals from 20 earlier simulations in which sequential segmentation evolved without the pressure to stop growing; then we continue their evolution in the presence of this pressure. The outcome of these continued simulations depends on the strength of the evolutionary pressure to stop growing. If the pressure is too low, determinate growth does not evolve often (Fig. 8b). If instead the pressure is too high, the potential for growth and segmentation is often transiently lost after which a simultaneous mechanism evolves instead; we do not observe smooth transitions from sequential to simultaneous segmentation (Fig. 8b). However, between these two extremes lies a parameter region in which one-third of simulations evolve the capacity to stop growing while maintaining posterior growth and sequential segmentation (Fig. 8b). Thus, our hypothesis is confirmed, delaying selection for determinate tissue growth to a later evolutionary stage does indeed more often yield the evolution of sequential growth and segmentation combined with determinate growth.

**Fig. 9:**
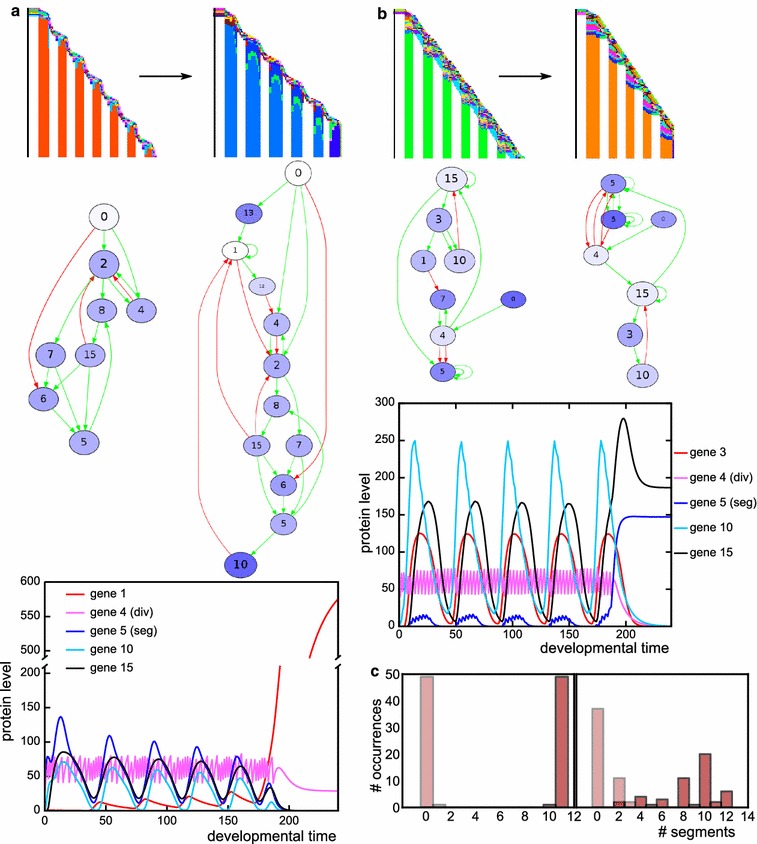
Mechanisms to stop growing differ in phenotypic variability (persistent signal, no CCS). **a**, **b** Individuals capable of sequential segmentation that are subsequently subjected to a pressure to stop growing. **a** This individual stops growing by increasing the expression of gene 1 over time. Note the addition of the extra module for stopping growth in the network. **b** This individual stops growing because gene 5 stochastically passes a threshold, above which its expression stabilises and switches off the division gene. **c** The individual of **a** maintains its developmental robustness (*left histogram*) and the one in **b** does not (*right histogram*). Both had very low phenotypic variation before the addition of the extra selection pressure, looking like the *left histogram*

In some of the cases where determinate growth evolves, the functional gene regulatory network expands to include a control gene. The expression of this control gene slowly increases over time until it passes a threshold and shuts down the division gene (Fig. 9a). This becomes evident in the different gene expression pattern in the last segment (Fig. 9a). More often, however, the stopping mechanism relies on an oscillating gene that passes a threshold due to the slight stochasticity in divisions and shuts down the division gene (Fig. 9b). This latter mechanism yields large phenotypic variability, while the former mechanism is more reliable (Fig. 9c).

Developmental programmes incorporating the first stopping mechanism (gene expression build-up) become slightly less robust compared to the original sequential segmentation mechanism without stopping growth, as stochastic divisions may influence the time at which the growth stopping protein level is being reached (Fig. 9c). Developmental programmes applying the second mechanism (stochastic threshold passing) become significantly less robust, which logically follows from the fact that they rely on the stochasticity of divisions to determine when to stop (Fig. 9c). Note that in both cases segment size does remain regular.

## Discussion

**Fig. 10:**
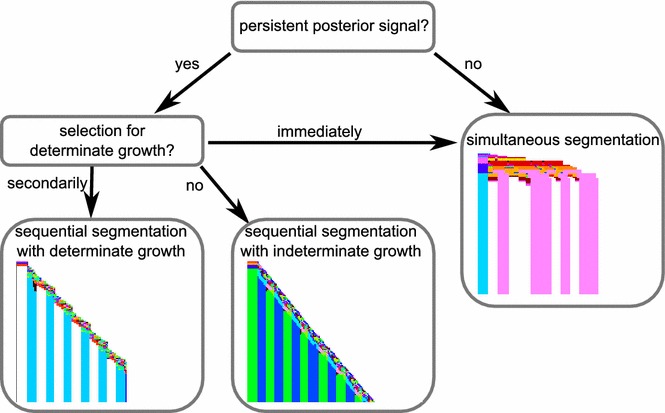
Summary of conclusions. Flowchart summarising the results of different simulations. Note that the *arrows* only indicate the majority of simulations in a set, e.g. persistent posterior signal without determinate growth selection occasionally yields simultaneous growth as well

A number of previous modelling studies have looked into the evolution of segmentation (see [36] for review) [24, 25, 29]. These studies mainly focused on the evolution of sequential segmentation (as in vertebrates or short-germ insects) versus simultaneous segmentation (long-germ insect, *Drosophila*-like), investigating their similarities and suggesting potential evolutionary transitions. In the current study, we took a somewhat different approach, focusing on factors that may have contributed to the likelihood of evolving sequential segmentation. We aimed to explain its dominance as a segmentation mechanism and the order of events through which it arose. Taking a “worse-case approach”, we maximally allowed alternative mechanisms to evolve and then determined under which conditions posterior growth and sequential segmentation predominates (Fig. 10).

We found one main alternative developmental strategy besides sequential segmentation: simultaneous segmentation, in which after a short tissue-wide burst of divisions all segments appears roughly at the same time. This simultaneous mechanism is not similar to *Drosophila*-like segmentation, where a hierarchy of gene regulation robustly creates regular-sized segments. Note that the *Drosophila* strategy likely evolved secondarily, from an initial sequential segmentation mode, so one should neither aim nor expect a *Drosophila*-like segmentation to evolve from scratch in our simulations. Rather, the evolution of a quite different type of simultaneous segmentation in our simulations is a result of the freedom of the evolutionary process, which we use to distinguish evolutionary scenarios. The simultaneous strategy that evolved in our simulations generates irregularly sized segments, and the number of segments in genotypically identical individuals tends to be variable. In contrast, the evolved sequential segmentation generates a large number of regularly sized segments in a robust, reproducible manner, thus leading to larger fitness values at the end of evolutionary simulations. A number of subtypes of posterior growth and sequential segmentation evolved; the most notable mechanism involves regular, segmental oscillation-dependent bursts of cell division in which two segments are down simultaneously—another benefit of the larger degrees of freedom of the model.

*Stable posterior signalling is a prerequisite for sequential segmentation* We showed that evolution of terminal addition type posterior growth is highly unlikely in the absence of persistent posterior signalling, independent of whether we selected for segmentation or body axis elongation. Under these conditions, the potential for symmetry breaking is restricted to the early phases of development, generating a bias in favour of an early tissue-wide burst of divisions and against posterior growth. In the absence of cell–cell signalling, simulations relied on the stochasticity of divisions to generate segments. In the presence of cell–cell signalling, lateral inhibition type patterning is used to pattern segments during the tissue-wide division burst. Our results thus suggest that the evolution of a posterior signalling centre is a crucial prerequisite for the evolution of posterior growth and sequential segmentation. Given the presence of a posterior signalling centre in all bilaterians as well as cnidarians, it can be safely assumed to represent an ancestral property [27, 37, 38]. Thus, we can reformulate our findings and state instead that the prior evolution of a posterior signalling centre provided a strong bias towards the evolution of posterior growth and sequential segmentation.

*Determinate growth as a secondary trait* In simulations incorporating a persistent posterior morphogen signal, selection for determinate growth completely reversed the evolutionary bias from sequential to simultaneous segmentation. We found that to evolve posterior yet determinate growth, the selection for determinate growth had to occur secondarily, after the evolution of posterior growth and sequential segmentation. Simple segmented animals such as millipedes and annelids contain large numbers of highly similar segments, and many annelids appear to keep adding segments throughout their life [18]. In contrast, insects and vertebrates develop a smaller, constant number of highly specialised segments after which posterior growth is terminated. We thus hypothesise that a constant segment number evolved secondarily and was only selected for once segment specialisation arose and locomotive capabilities became restricted to a limited number of segments. Consistent with this, HOX genes, which are crucial in segment specialisation, appear to be involved in terminating posterior growth [39]. As an intermediate form, myriapods and the extinct trilobites stop adding segments when reaching maturity, but the final number of segments is variable [9]. This has been linked to their limited segment specialisation, where the exact number of segments is not that important. This is reminiscent of the form of determinate growth that evolves in our simulations, which is not robust and yields variable segment numbers.

Still, how to explain the fact that many unsegmented and metameric animals display determinate growth, for instance *C. elegans*? Assuming an unsegmented bilaterian ancestor, determinate growth may have evolved prior to sequential segmentation. In the current study, we did not explicitly test for this; however, we expect that even if we selected for only axial tissue growth without segmentation, simultaneous growth would arise if we also immediately selected for determinate growth. Given that ancestral state reconstructions suggest that terminal addition is an ancestral bilaterian trait [9, 14] while the evidence is less conclusive for sequential segmentation, we would expect that determinate growth at least evolved secondary to posterior growth. Alternatively, the presence of determinate growth in unsegmented and metameric organisms can be explained by the presence of a bilaterian ancestor displaying terminal addition, sequential segmentation and determinate growth with many lineages subsequently completely or partly losing segmentation. Finally, a less parsimonious scenario involves an unsegmented, indeterminately growing bilaterian ancestor, with parallel evolution of either determinate growth alone, or following sequential segmentation in several lineages.

In our current model we observed two mechanisms to stop posterior growth: one depending on stochastic changes in an oscillating gene, making it very non-robust, and the other depending on the gradual build-up of a slowly decaying gene, yielding lower phenotypic variability. This latter strategy resembles a hypothetical mechanism proposed by Meinhardt for the sequential activation of HOX genes [30, 40]. Considering the origin of the HOX cluster from tandem duplication of an early HOX gene [41], it can be envisioned that an early gene involved in regulating growth became transformed through duplication into a sequentially activated HOX cluster in which gene order (i.e. posterior-most HOX gene active) rather than level of a single gene now can act as a robust growth termination criterion. An interesting subject for future studies would thus be to investigate whether under combined selection for both segmentation and HOX-like specialisation domains, a HOX-type control of growth evolves. Such evolutionary outcomes may provide important answers in the puzzle of how the complex hierarchical network of Drosophila evolved from a sequentially segmenting predecessor; given the relatedness of gap and HOX genes and the suggested ancestral role for gap genes in growth control.

## Conclusions

In summary, we proposed an order of evolutionary events and selection pressures involved in generating posterior growth, sequential segmentation and determinate growth. First, we provided evidence that the prior evolution of a stable posterior signalling centre has played a decisive role in evolving terminal addition and sequential segmentation. Then, we showed that the evolution of sequential segmentation combined with determinate growth can only take place by adding the selection pressure for determinate growth secondarily. Our study demonstrates that varying the onset of selection pressures can be a powerful tool in investigating the likely order of evolutionary events.

## Additional files

10.1186/s13227-016-0052-8 Cell-cell signalling allows for robust development in the absence of posterior morphogen (but not always) (transient signal, CCS). A) This individual evolved to be very robust. It usually makes eight segments and only in rare cases seven good segments and one short segment. B) This individual did not evolve to become very robust despite the presence of cell–cell signalling genes. Nevertheless, it is usually able to make many more segments than the individuals evolved without cell–cell signalling. The histograms represent the variation in phenotypic outcome when an individual{\rsquo}s development is repeated 50 times. In the histograms, the dark bars represent the good segments and the lighter bars the too short segments. See “Methods” for further explanation.
10.1186/s13227-016-0052-8 Very rare evolution of posterior growth in the absence of persistent posterior signal (CCS, no noise) A) This individual first creates a small pool of five cells in an initial burst, which subsequently become completely inert (the black cells in the left space–time plot). The sixth cell, which is still in contact with the head, gets induced via CCS (gene 1, circled in yellow in the network) to initiate another burst of divisions, and these cells form the first two segments (high -> green; and low -> blue) next to the head (see cartoon). When these new segments mature, there is a short time window in which the most anterior of the five posterior inert cells gets induced to initiate a new burst. Thus, the posterior pool gets depleted by one cell with each burst, putting a stop to the growth process. (Note the shortening of the dark, non-dividing region in the space-time plots) In the network, the division gene is circled in magenta and the segmentation gene in blue. B) This mechanism arises very late in evolution, from a simultaneously segmenting individual.
10.1186/s13227-016-0052-8 Simultaneous segmentation with persistent posterior morphogen can be more robust than with transient signal (no CCS) A comparison between transient and persistent posterior morphogen on the evolved simultaneous mechanisms. Those evolved with persistent signalling are capable of making more segments and are sometimes very robust. When they are not robust, they still manage to make more segments on average. Note that the second individual with transient signalling may be robust, but this means it usually makes just two segments.
10.1186/s13227-016-0052-8 Two examples of non-robust development with sequential segmentation (persistent signal, no CCS) Top row, developmental space-time plots; second row, histogram of the outcome of 50 repeated developments; third row, evolved gene interaction networks and examples of variation in segmentation gene expression; fourth row, gene expression in the posterior cell with normal divisions; bottom row, gene expression in the posterior cell with averaged division gene expression instead of divisions. A) The gene expression oscillations of this individual are entirely dependent on the regular divisions (oscillations are absent when the division gene is averaged), and are therefore very sensitive to division noise. B) While the oscillations in this individual do not depend on the divisions themselves, they are influenced by the level of the division gene. Stochastic changes in the timing of division result in changes in the level of the division protein (see circled point in the graph) which may alter the fate of the daughter cell emanating from the growth zone.

## Abbreviations

CCS: cell–cell signalling
TF: transcription factor
TFBS: transcription factor binding site
