# Supplementary figures and images for: In silico evo-devo: reconstructing stages in the evolution of animal segmentation
Source: EvoDevo. 2016 Aug 1;7:14. doi: 10.1186/s13227-016-0052-8 (PMC4968448; doi:10.1186/s13227-016-0052-8)

**A**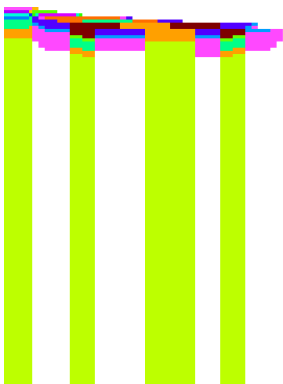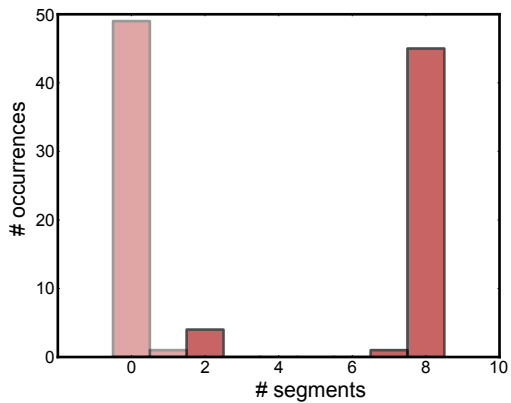**B**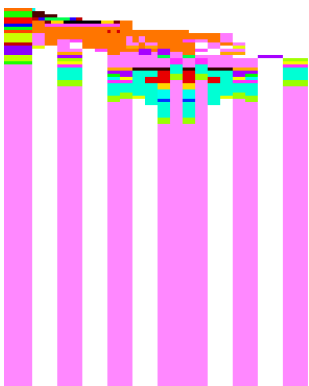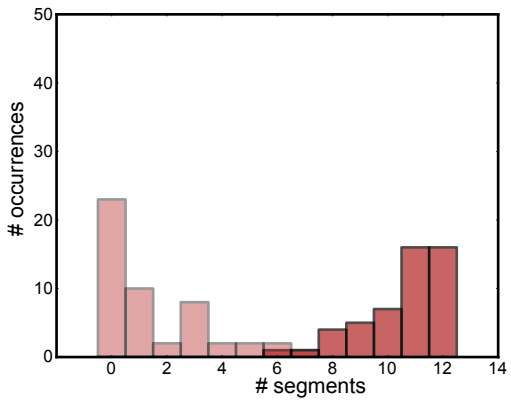

Supplement: Supplementary file 1 — 10.1186/s13227-016-0052-8 Cell-cell signalling allows for robust development in the absence of posterior morphogen (but not always) (transient signal, CCS). A) This individual evolved to be very robust. It usually makes eight segments and only in rare cases seven good segments and one short segment. B) This individual did not evolve to become very robust despite the presence of cell–cell signalling genes. Nevertheless, it is usually able to make many more segments than the individuals evolved without cell–cell signalling. The histograms represent the variation in phenotypic outcome when an individual{\rsquo}s development is repeated 50 times. In the histograms, the dark bars represent the good segments and the lighter bars the too short segments. See “Methods” for further explanation. [file 13227_2016_52_MOESM1_ESM.pdf]

**A**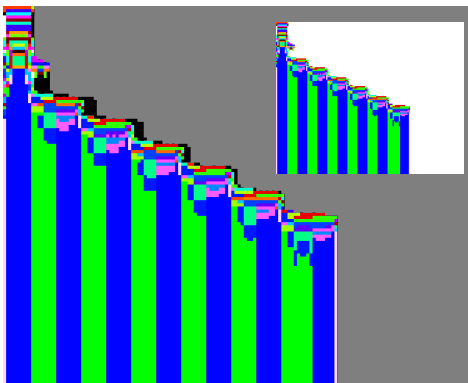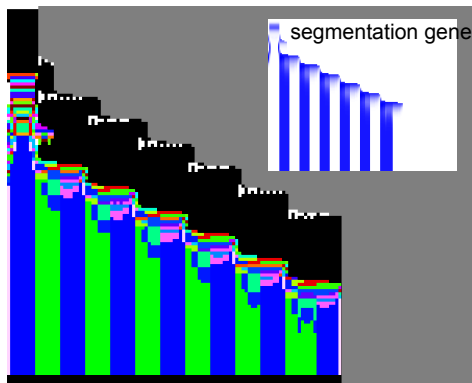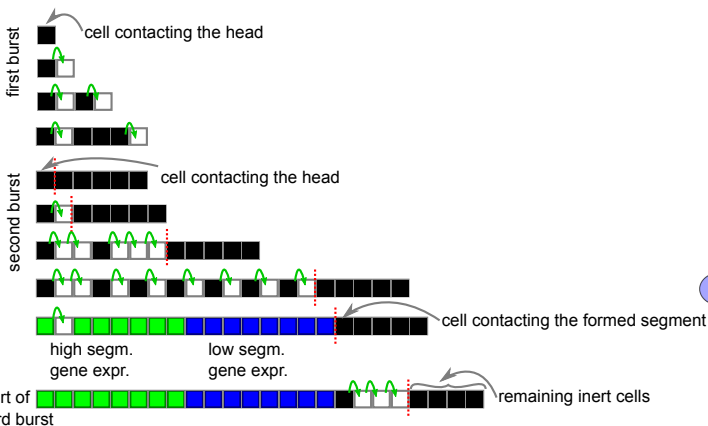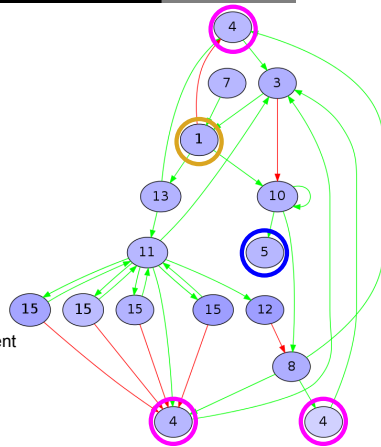**B**

evolutionary time: 48400

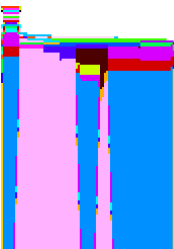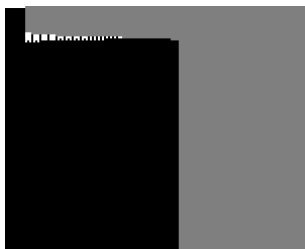

evolutionary time: 48500

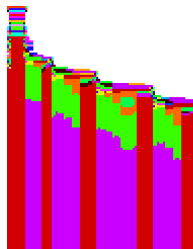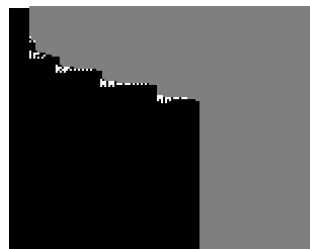

evolutionary time: 48600

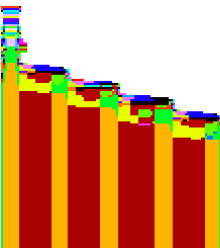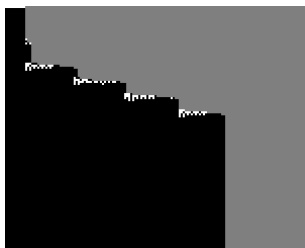

evolutionary time: 48700

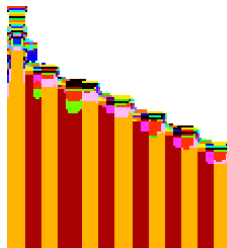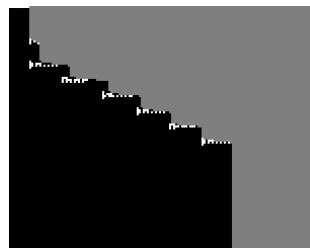

Supplement: Supplementary file 2 — 10.1186/s13227-016-0052-8 Very rare evolution of posterior growth in the absence of persistent posterior signal (CCS, no noise) A) This individual first creates a small pool of five cells in an initial burst, which subsequently become completely inert (the black cells in the left space–time plot). The sixth cell, which is still in contact with the head, gets induced via CCS (gene 1, circled in yellow in the network) to initiate another burst of divisions, and these cells form the first two segments (high -> green; and low -> blue) next to the head (see cartoon). When these new segments mature, there is a short time window in which the most anterior of the five posterior inert cells gets induced to initiate a new burst. Thus, the posterior pool gets depleted by one cell with each burst, putting a stop to the growth process. (Note the shortening of the dark, non-dividing region in the space-time plots) In the network, the division gene is circled in magenta and the segmentation gene in blue. B) This mechanism arises very late in evolution, from a simultaneously segmenting individual. [file 13227_2016_52_MOESM2_ESM.pdf]

transient posterior signalling

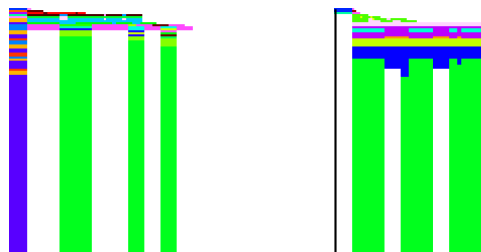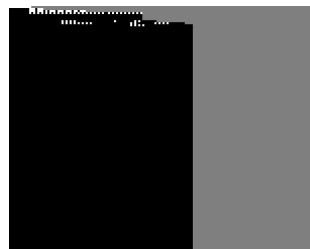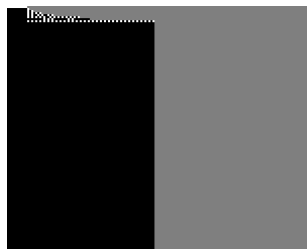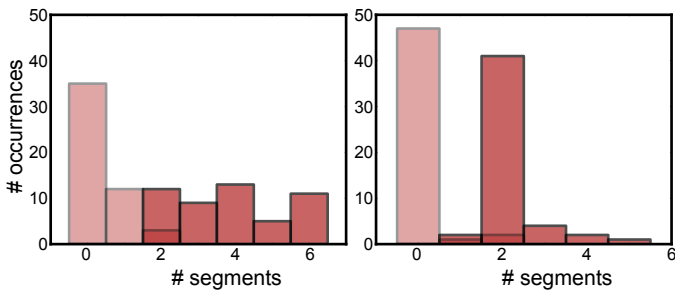

persistent posterior signalling

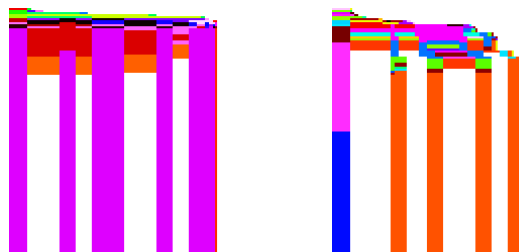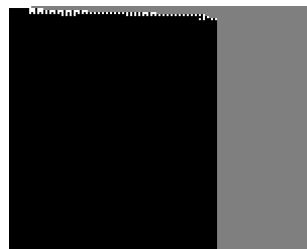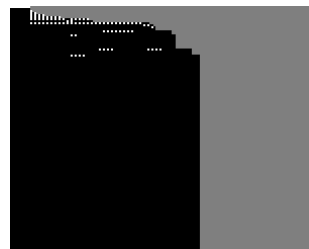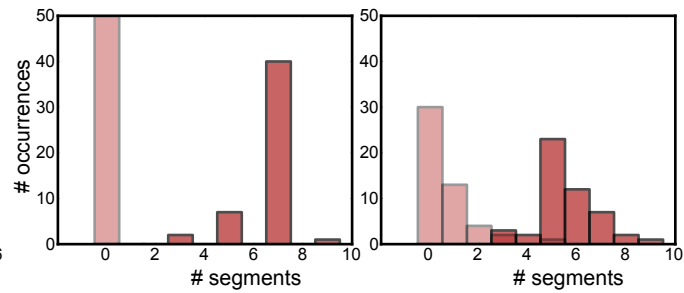

Supplement: Supplementary file 3 — 10.1186/s13227-016-0052-8 Simultaneous segmentation with persistent posterior morphogen can be more robust than with transient signal (no CCS) A comparison between transient and persistent posterior morphogen on the evolved simultaneous mechanisms. Those evolved with persistent signalling are capable of making more segments and are sometimes very robust. When they are not robust, they still manage to make more segments on average. Note that the second individual with transient signalling may be robust, but this means it usually makes just two segments. [file 13227_2016_52_MOESM3_ESM.pdf]

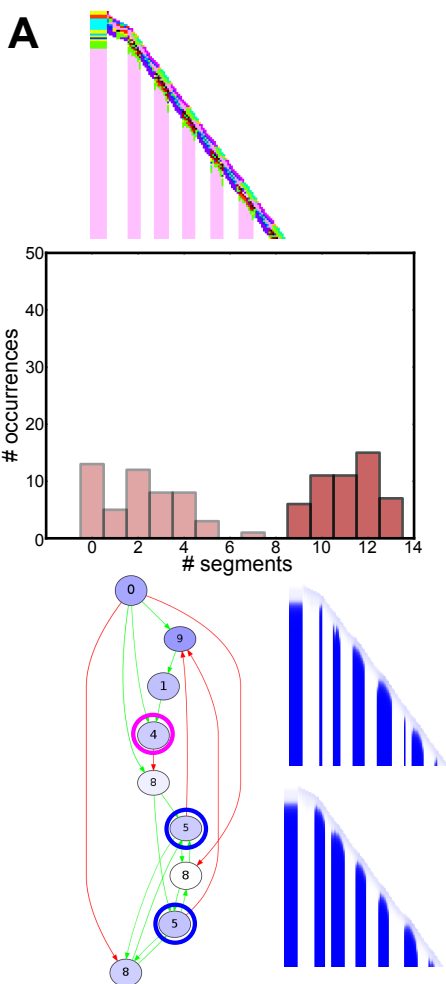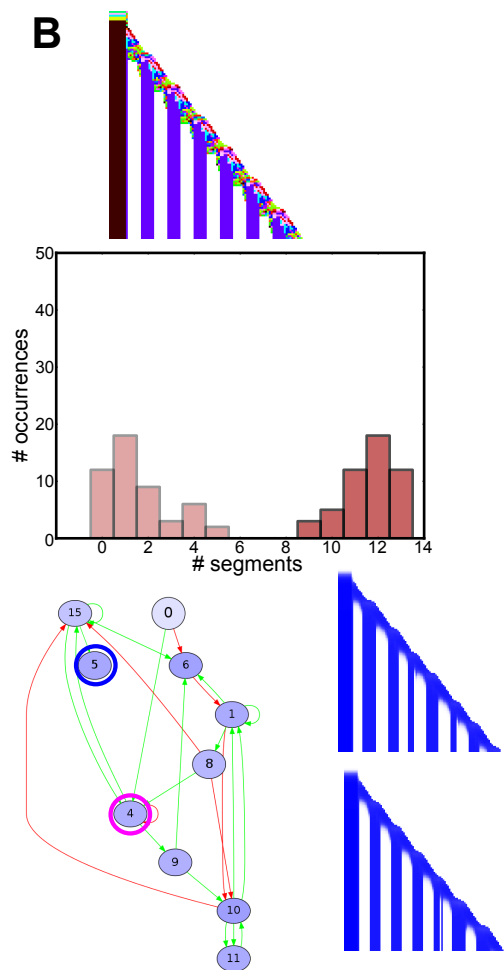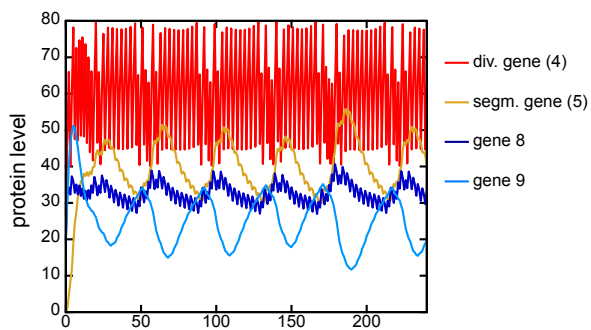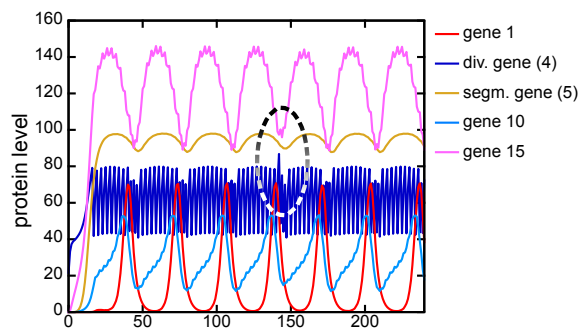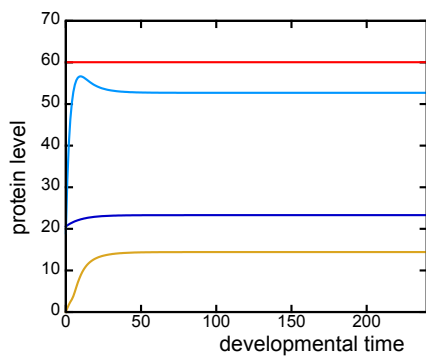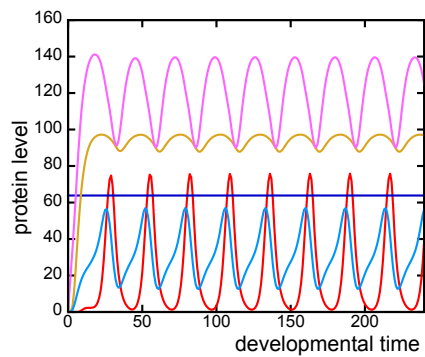

Supplement: Supplementary file 4 — 10.1186/s13227-016-0052-8 Two examples of non-robust development with sequential segmentation (persistent signal, no CCS) Top row, developmental space-time plots; second row, histogram of the outcome of 50 repeated developments; third row, evolved gene interaction networks and examples of variation in segmentation gene expression; fourth row, gene expression in the posterior cell with normal divisions; bottom row, gene expression in the posterior cell with averaged division gene expression instead of divisions. A) The gene expression oscillations of this individual are entirely dependent on the regular divisions (oscillations are absent when the division gene is averaged), and are therefore very sensitive to division noise. B) While the oscillations in this individual do not depend on the divisions themselves, they are influenced by the level of the division gene. Stochastic changes in the timing of division result in changes in the level of the division protein (see circled point in the graph) which may alter the fate of the daughter cell emanating from the growth zone. [file 13227_2016_52_MOESM4_ESM.pdf]
